# One Good Deed

**Published:** 1885-11

**Authors:** 


### One Good Deed.

Bill Poole, of New York, sport and pugilist, whose murder by Lewis Baker years ago created such excitement, was not a model man. He did not pretend to be, and while so pretending rob his fellows. He had some generous traits which may be reckoned up hereafter. It is related of him that one Thanksgiving day, just before he was killed, he came to a marketman's stand and putting down $100 said: "Send chickens and turkeys for that amount to the charities." "You can't have all of this," the dealer replied. "We mean to have a hand in." The result was the poultry dealers of the market clubbed together, and the next day all the principal charities of New York received turkeys and chickens without stint. Bill Pool's $100 was only a drop in the bucket, but it was the drop that filled it. Since that time Washington Market has contributed tons of poultry and meat to the charities of the city, and has organized a system that aids materially in the efforts of the charitable to make at least one day of the year a pleasant and happy one to the unfortunate.

This shows how one good action often impels many, and it demonstrates, too, that, among the outcast of the earth, there is often a flicker of the divine spark that redeems them from total depravity.

Harness Blacking.—In an old scrap-book I find this recipe which is said "will give a beautiful polish:" 2 ozs. white wax and 3 ozs. of turpentine are to be dissolved together over a slow fire; then add 1 oz. ivory-black and 1 drachm indigo, to be well pulverized and mixed together. "When the wax and turpentine are dissolved, add the ivory-black and the indigo, and stir till cold." Apply very thin and brush afterwards.

Barley Water.—I have found this useful in private practice to keep up the strength of a patient: A cupful of barley in two quarts of water, allowed to boil two hours. About an hour after it is on the fire, add a dozen stoned raisins; strain before serving. If I wish a stimulant, I put in the glass or cup in which I serve it a teaspoonful of brandy or sherry wine. It ought to be cooked in a porcelain-lined vessel or it is apt to discolor.

